# A contribution to the knowledge of the mountain entomofauna of Mexico with a description of two new species of *Onthophagus* Latreille, 1802 (Coleoptera, Scarabaeidae, Scarabaeinae)

**DOI:** 10.3897/zookeys.572.6763

**Published:** 2016-03-15

**Authors:** Victor Moctezuma, Michele Rossini, Mario Zunino, Gonzalo Halffter

**Affiliations:** 1 Red de Ecoetología; Instituto de Ecología A.C. – Carretera antigua a Coatepec, 351 – 91070 XALAPA (VER) – México; 2 Università di Urbino Carlo Bo, Dipartimento di Scienze Biomolecolari (DISB), via A. saffi, 2, 61029, Urbino, Italia; 3 Universitá di Urbino Carlo Bo, Dipartimento di Scienze della Terra, della Vita e dell’Ambiente (DiSTeVA), Campus Scientifico “Enrico Mattei”, via Ca´ Le Suore, 2, 61029 URBINO (PU), Italia; 4 Universidade Federal de Mato Grosso, Instituto de Biociências, Departamento de Biologia e Zoologia, Av. Fernando Corrêa da Costa, 2367, Boa Esperança, 78060-900 - Cuiabá, MT, Brazil

**Keywords:** Scarabaeinae, *Onthophagus*, mountain fauna, Mexican Transition Zone, Trans-Mexican Volcanic Belt

## Abstract

Recent intensive samplings carried out across the mountainous regions of El Pinal (Puebla, Mexico) have provided new insights into the main environmental factors that affect the geographic distribution of the scarabaeinae beetles of the Trans-Mexican Volcanic Belt above 2500 m a.s.l. This study is part of an ongoing project investigating the diversity and biogeography of copro-necrophagous beetles (Scarabaeinae, Aphodiinae, Geotrupinae and Silphidae) in the easternmost areas of the Trans-Mexican Volcanic Belt.

Previous experience allows us to propose a series of predictions that we expect will provide possible explanations for current distribution patterns observed in Scarabaeinae and other groups of insects found in the Trans-Mexican Volcanic Belt. This mountain range has a primarily biogeographic importance, limiting the Mexican High Plateau in the South and connecting the western and eastern Sierra Madre mountain chains, which are considered the most important routes for dispersal of mountain fauna of northern origin. The taxonomic and biogeographic study of the species collected so far in El Pinal (including *Onthophagus clavijeroi* **sp. n.** and *Onthophagus martinpierai* **sp. n.** described here), along with their possible relationships with other known species, allows us to answer the preliminary assumptions proposed.

## Abstract (es)

Con base en capturas intensivas realizadas en la montaña de El Pinal (estado de Puebla, México) se plantea un análisis de los factores que intervienen en la distribución geográfica de la fauna de montaña (en este caso Scarabaeinae) por encima de los 2500 m de altitud, en el Sistema Volcánico Transversal (SVT), principal cordillera dispuesta en sentido latitudinal de México y de toda América. Este artículo es parte de una serie de trabajos coordinados para estudiar los escarabajos coprófagos y necrófagos de la parte oriental del SVT.

La experiencia generada nos permite plantear una serie de premisas (Introducción) que se espera cumpla la distribución de la fauna de Scarabaeinae y de otros grupos en el SVT, cordillera de primera importancia biogeográfica, pues limita por el sur el Altiplano Mexicano (una península de Norte América) y une las dos principales cordilleras que se desarrollan N-S: las Sierras Madres Occidental y Oriental, las más importantes vías de penetración de la fauna de montaña de origen septentrional. Un análisis de las afinidades taxonómicas y biogeográficas de las colectas de Scarabaeinae en El Pinal (incluyendo dos especies nuevas de *Onthophagus* que se describen) nos permite dar respuesta en la Discusión a las premisas planteadas en la Introducción.

## Introduction

Mexico is a country of vast mountain ranges. In tropical areas, these environmental conditions imply a rich and peculiar biota; the particular geographic position of Mexico and the extensive Mexican Transition Zone, which occurs between the Nearctic and Neotropical regions, emphasizes the biological exclusivity of this region.

The Mexican Transition Zone (hereafter referred to as MTZ) has been proposed by Halffter for insects and other groups of animals (e.g., Halffter 1987; Halffter 2003; Halffter et al. 2008), while Rzedowski introduced the concept of Megamexico, a phytogeographic unit defined on the basis of distribution patterns observed in Mexican plants (Rzedowski 1991). The concept of the transition zone has also been utilized in general terms by Zunino and Zullini (2003: 66–68 and literature quoted therein). A biogeographical transition zone is a geographical area of biotic juxtaposition with a gradient of replacement and partial segregation between components, which are promoted by historical and ecological changes. During his travel across the Malay Peninsula between 1854 and 1862, A. R. Wallace had already realized the difficulty of delimiting clear biogeographical borders between the Oriental and Australian regions, a wide area with transitional characteristics, which is today known as Wallacea (see Ferro and Morrone 2014 for a very interesting and updated revision of biogeographic transition zones).

The transition occurs when biota of two different regions, the distribution areas of which have not been historically constrained by significant biogeographical barriers, meet and overlap within the same geographic area and time. If the transition character remains temporally, this zone may harbour species of different geographical and evolutionary origins.

**Map 1.**
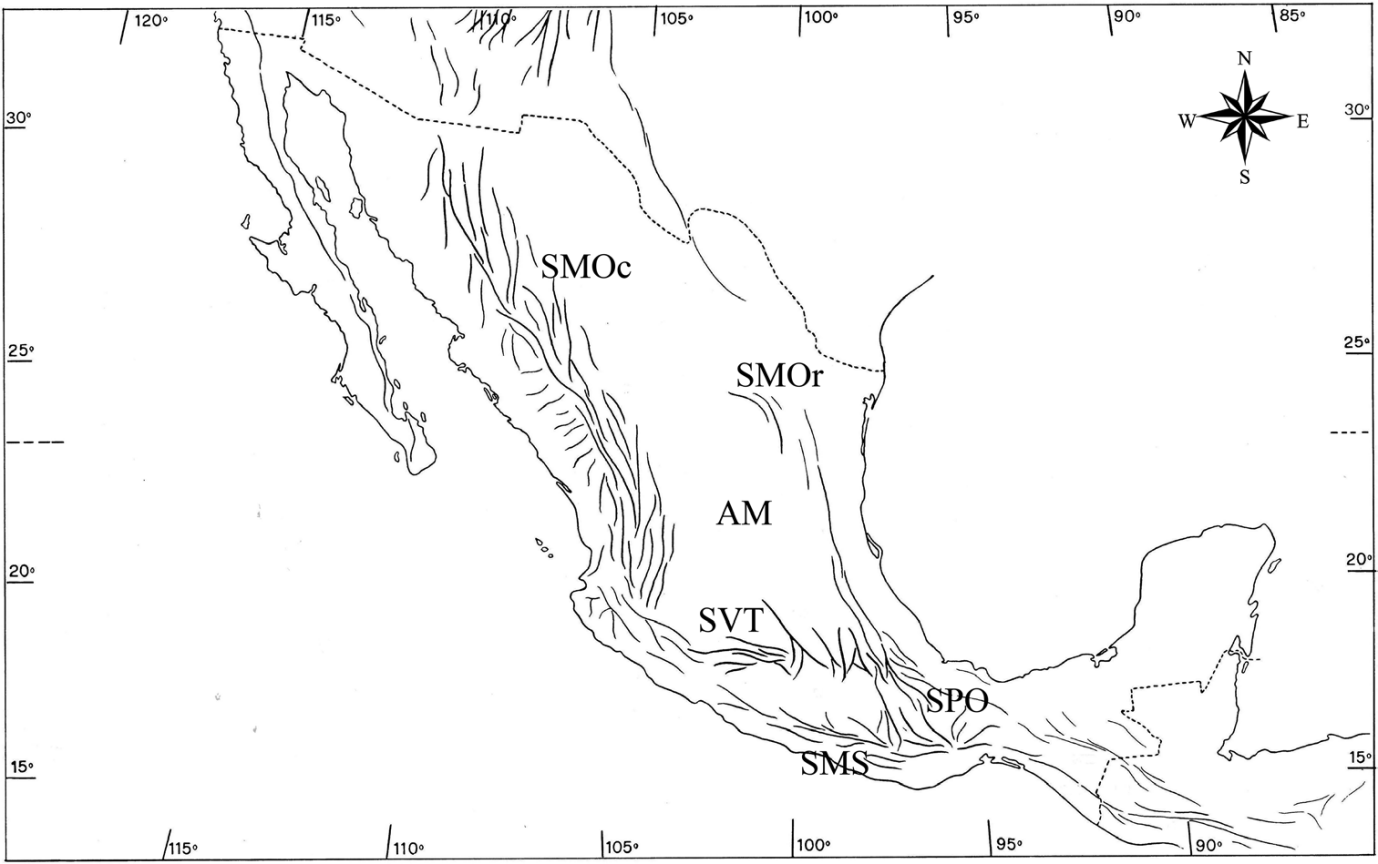
Orographic systems mentioned in the text: SMOc: Sierra Madre Occidental; SMOr: Sierra Madre Oriental; AM: Mexican Plateau; SVT: Trans-Mexican Volcanic Belt; SMS: Sierra Madre del Sur; SPO: Puebla-Oaxaca Mountain System.

The mountain ranges of the MTZ play a fundamental role as a main route for the southward dispersal of northern fauna. Among the Mexican mountain ranges, the Trans-Mexican Volcanic Belt (hereafter referred to as TMVB) is the only one in the Americas with latitudinal development, intervening as central bridge between the western and eastern Sierra Madre mountain ranges (see Map 1).

Recently, Luna et al. (2007) provided a description of the geology, physical environment, as well as flora and fauna of the TMVB, presenting several biogeographic studies.

The TMVB features a series of mountain ranges and volcanoes that cross Mexico transversally between the parallels 18°30’ and 21°30’ W. The orogenesis of this area is widely accepted to result from the subduction of both the Rivera Microplate and northern border of the Cocos Plate under the southern boundaries of the North American Plate. Since the Early-Mid Miocene, the TMVB became an independent geological province because of the East-West rotation of the Transvolcanic Belt, as well as the change of type and composition of the main volcanic activities: silicious and explosive in the Sierra Madre Oriental, intermediate and eruptive in the TMVB (Ferrari et al. 2012).

Four main volcanic events that occurred over the last 19 million years contributed primarily to the formation of the TMVB, with the greatest volcanic activity recorded in the Quaternary. This volcanic eruption also involved the areas and locations investigated in this study, such as the mountains Pico de Orizaba, Cofre de Perote and La Malinche (see Torres-Miranda and Luna 2007 and other chapters in Luna et al. 2007).

The TMVB stretches for approximately 1000 km in length and, despite its current unity, the geological history of the areas lying east and west of the meridian 101° does not show any degree of continuity until the end of the Pliocene and Quaternary. Along with the geomorphology of the TMVB, the main stratovolcanoes of the central and eastern regions built up in the Upper Pleistocene (see Ferrari et al. 2012) are of great importance for explaining several biogeographic patterns, as well as vicariant events that occurred along the TMVB and are commonly represented within mountain lineages of northern origin. Initially, the lineages found only either in the western or eastern halves of the TMVB can be considered to be the oldest, with different biogeographic histories, as these are related to the Sierra Madre Occidental, the Sierra Madre del Sur or the Sierra Madre Oriental, respectively. On the other hand, lineages or species that are widely distributed across the entire TMVB can be considered the most recent ones, and show a succession of very closely related taxa.

Given its East-West orientation (Map 1) and dynamic geomorphological history, the TMVB is not only an important dispersal route for northern fauna with mountain adaptations, but is also an area characterized by a large number of vicariant events reported for many organisms as well as a high beta diversity (see Luna et al. 2007).

All of the results obtained to date are included in seven articles (including the present study), (Sanchez-Huerta 2015, Arriaga et al. 2016, Moctezuma et al., Skelley and Sanchez-Huerta, Sanchez-Huerta et al. A, Sanchez Huerta et al. B), two Master’s dissertation (Moctezuma 2014, Sanchez-Huerta 2016) and a Doctoral thesis (Arriaga 2015).

The initial predictions to be tested throughout these studies are as follows:

The entomofauna (especially Scarabaeinae) of the TMVB above 2500 m a.s.l. corresponds to the Paleo-American Distribution Pattern (see Halffter 1987, Halffter 2003, Halffter et al. 2008), that are northern lineages belonging to worldwide distributed genera with old penetration in the American continent. Within this distributional pattern, the Mountain Paleo-American sub-pattern, which includes lineages limited to the mountains of the MTZ, should take over.

The dispersal of lineages of the Mountain Paleo-American distribution pattern essentially occurs horizontally (Lobo and Halffter 2000), following a given altitudinal range and presenting dispersal events only under advantageous climatic conditions, whereas isolation and vicariant events appear under adverse climatic conditions, which are also responsible for the reduction of the area of distribution. Time represents a basic difference between horizontal and vertical colonization; vertical colonization requires the adaptation of lineages to new environmental conditions while horizontal colonization only requires dispersal.

The bioclimatic zones in mountainous areas experienced major altitudinal shifts during the Quaternary and Recent periods. The complex orography of the MTZ, especially that of the TMVB, along with the catathermal climatic periods, had strong effects on the high-altitude communities, which underwent a severe displacement toward the lowlands that were interconnected to a greater extent. Under the new environmental conditions, these biological elements found a favourable situation in which to spread horizontally. However, during anathermal periods, bioclimatic zones move upward to higher elevations, with a consequent fragmentation of the distribution area of the species involved. These events are often followed by speciation processes associated with dynamic vicariance (see Zunino 2003: 161; Morrone 2009: 16).

Occasionally there are cases of vertical colonization, represented by upward dispersal from the lowlands. This dispersal event is expected to occur in species with the Paleo-American High Plateau Distribution sub-pattern (Halffter 2003). In some exceptional cases, some species of Neotropical affinity distributed on the Mexican High Plateau might penetrate the mountains, particularly at lower elevations.

Vertical movements are not expected on the southern slopes of the TMVB such as the Balsas and Tehuacán-Cuicatlán depressions, the fauna of which, adapted to warm and dry conditions, shows Neotropical affinity. The rare antiquity of the mountain El Pinal, but particularly the strong contrast in ecological conditions, has not favored the upward displacement of tropical species.

The dispersal-distribution pattern with predominantly horizontal distribution, observed in the TMVB and in Mexican mountain ranges in general, is completely different from the pattern found in the Andes (see Escobar et al. 2006, 2007) or on the Mexican mountains that are isolated within a tropical environment, such as Los Tuxtlas (Alvarado-Roberto 2014). In these cases, vertical colonization appears to be the more common distribution pattern, with the Neotropical fauna moving toward higher altitudes from the tropical lowlands.

In these tropical mountains with vertical colonization, the highest species richness occurs at intermediate elevations (at approximately 1500 to 1700 m a.s.l.), with rarefaction occurring at higher altitudes (Escobar et al. 2007). In the TMVB, which is colonized by fauna of northern origin, there is a representative number of species exclusive to high elevations that are not found at lower altitudes.

The species richness of Scarabaeinae on tropical mountains with vertical colonization is higher in wooded areas, as occurs in the surrounding lowlands (Escobar 2004, Escobar et al. 2007). As in Europe (see for example Errouissi et al. 2004, Jay-Robert et al. 2007), a higher number of Scarabaeinae species occur in opened or semi-opened areas in the TMVB, with a very low presence of species characteristic of forests and woodlands.

In this article, we describe two new *Onthophagus* species collected to date only in the westernmost mountains of the eastern sector of the TMVB (El Pinal). Phylogenetic and biogeographic relationships of both species are discussed, and our preliminary hypotheses on Scarabaeinae and other copro-necrophagous Scarabaeoidea of the TMVB are tested against the distribution patterns observed in these new *Onthophagus* and other species collected in the same sites.

## Material and methods

Sampling was conducted on the westernmost mountains of the eastern sector of the TMVB (El Pinal, Puebla, Mexico). To date, the entomofauna of this area has been little studied compared to the tropical sector of the Mexican Transition Zone.

Copro-necrophagous beetles were collected using common pitfall traps baited with human and cow excrements, and squid. Collected specimens were processed at the Institute of Ecology (INECOL) in Xalapa.

The morphological study was carried out on multiple specimens belonging to the new species described here. In order to study the male and female genital organs, individuals were softened in hot water for approximately 20 minutes and their genitalia extracted. In males, the aedeagus was dissected for examination of the endophallic sclerotized structures of the internal sac. Genital structures of both sexes were cleared in a 10% KOH solution. Finally, the aedeagus, endophallic sclerotized pieces and vagina were either mounted on a paper-board with DMHF resin or preserved in microvials with glycerol.

Specimens collected during fieldwork were deposited in the following private and public collections (information regarding the repositories of holotypes and paratypes are provided with the species descriptions):

CNC
 Canadian National Collection, Ottawa, Canada 


GH
 Gonzalo Halffter collection, Xalapa, Mexico 


INECOL
 Instituto de Ecología A. C., Xalapa, Mexico 


LD
 Leonardo Delgado collection, Xalapa, Mexico 


MM
 Miguel A. Morón, collection, Xalapa, Mexico 


MR
 Michele Rossini collection, Pesaro, Italy 


MZ
 Mario Zunino collection, Asti, Italy 


VM
 Victor Moctezuma collection, Xalapa, Mexico 


## Results

### Onthophagus clavijeroi

Taxon classificationAnimaliaColeopteraScarabaeidae

Moctezuma, Rossini & Zunino sp. n.

http://zoobank.org/74F35DE3-6094-48F3-948E-F0BB7992EA1D

#### Material examined.

Holotype: male pinned with genitalia in microvial. 1. Label Holotypus (in Spanish) “Mexico, El Pinal, Pue., at 0.3 km from Rincón, 2/VII/13, necrotrap 7n2, x- 97°53'59.8" W, y- 19°8'55.3", shrubby veg., 2704 m a.s.l., Moctezuma J.V.P. Col.” (1 ♂ GH). Paratypes: 1 ♂, same label as the holotype; 2 ♂♂, labeled “Mexico, El Pinal, Pue., at 0.3 km from Rincón, 2/VII/13, necrotrap 7n6, x- 97°54'1.1" W, y- 19°8'56.8", oak forest, 2710 m a.s.l., Moctezuma J.V.P. Col.”; 2 ♂♂, 1 ♀ labeled “Mexico, El Pinal, Pue., at 0.3 km from Rincón, 2/VII/13, necrotrap 7n7, x- 97°53'59.8" W, y- 19°8'54.3", oak forest, 2704 m a.s.l., Moctezuma J.V.P. Col.”; 1 ♀ labeled “Mexico, El Pinal, Pue., at 2.5 km from Rincón, 26/VI/13, necrotrap 5n1, x- 97°55'2.1" W, y- 19°7'46.6", oak forest, 2543 m a.s.l, Moctezuma J.V.P. Col.”.

#### Description.

**Figure 1.**
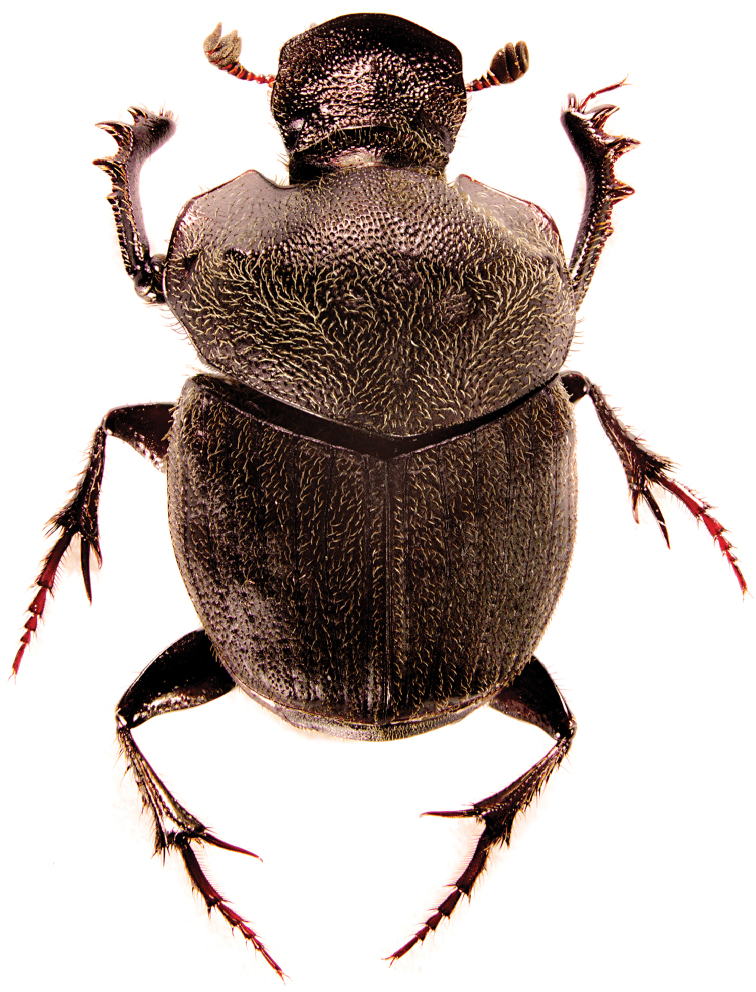
*Onthophagus clavijeroi* sp. n. major male.

Holotype. Major male (Fig. 1). Length 9.4 mm, maximum width of pronotum 5.3 mm. Body dark-brown and dull with cupreous casts, antennal club dark grey, body covered with yellow and light setae. Clypeus transversally developed and pentagonal shaped, distinctly concave, with anterior margin widely curved at middle. Head with lateral margins slightly sinuate in proximity of clypeo-genal suture, genae with margins subparallel, genal suture distinct. Clypeal carina absent to very feebly indicated, frontal carina subtrapezoidal with anterior convexity and elevated at middle with a triangular tubercle, frons coarsely punctured, with short and light-yellow setae, forward curved. Clypeus with strong and coarse punctures, punctuation almost confluent, some punctures with a long seta, few and shorter setae near the apex of clypeus. Posterior margin of pronotum with evanescent border, lateral margins straight to barely concave between anterior and median angles. Pronotal protuberance not regularly convex, pronotum distinctly sloped between median tubercles and anterior margin, pronotal protuberance obtusely trapezoidal, anterior tubercles absent, posterior tubercles strong and slightly forward than the middle of pronotum. Pronotal disc with irregular and coarse punctuation, punctures slightly elongated and crowded, pronotal surface between punctures clearly microsculptured, punctures with a long and light seta, pilosity shorter and scattered in proximity of apex of pronotal protuberance. Elytral striae impressed, bright and with medium-sized punctures well-spaced, interstriae flattened with finer punctuation than pronotum, every puncture bears a light-yellow seta, microsculpture evident. Pygidium dull and sericeous at the base, apically bright, pygidial surface with inconspicuous punctuation, punctures with a short and light seta, median and longitudinal part of pygidium bare and lacking significant punctures, microsculpture of pygidium reticular. Apex of pygidium with bigger, transversal and confluent punctures, microsculpture less evident. Foretibiae slender, distinctly curved and slightly wider at apex, apical spur with apex distinctly curved downward.

Parameres and endophallic sclerites: Figs 4–6.

Female (Fig. 3): Clypeus more elongated forward than in male, clypeal carina strong and evenly curved, frontal carina trapezoidal, lateral margins of clypeus straight, apex widely curved, clypeo-genal suture not indicated on lateral margin of the head. Lateral margins of pronotum curved, anterior pronotal protuberance ill-defined, disc of pronotum with two circular and flattened areas. Protibiae distinctly wider and less curved than in male. Female genitalia: Fig. 7.

**Figure 2.**
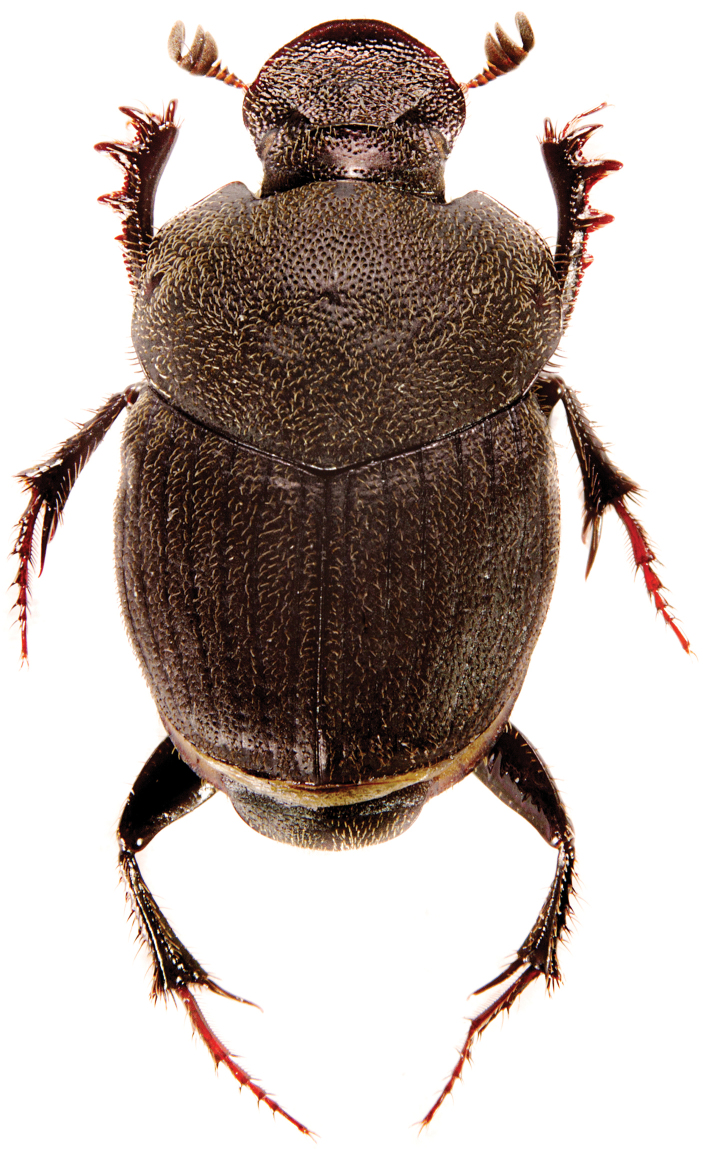
*Onthophagus clavijeroi* sp. n. minor male.

Variation. Minor male (Fig. 2): Smaller than male, clypeus more distinctly trapezoidal, clypeal carina very weakly indicated and slightly swollen at middle, frontal carina reduced and lacking median tubercle, anterior pronotal protuberance ill-defined to absent, disc with two flattened and circular areas.

**Figure 3.**
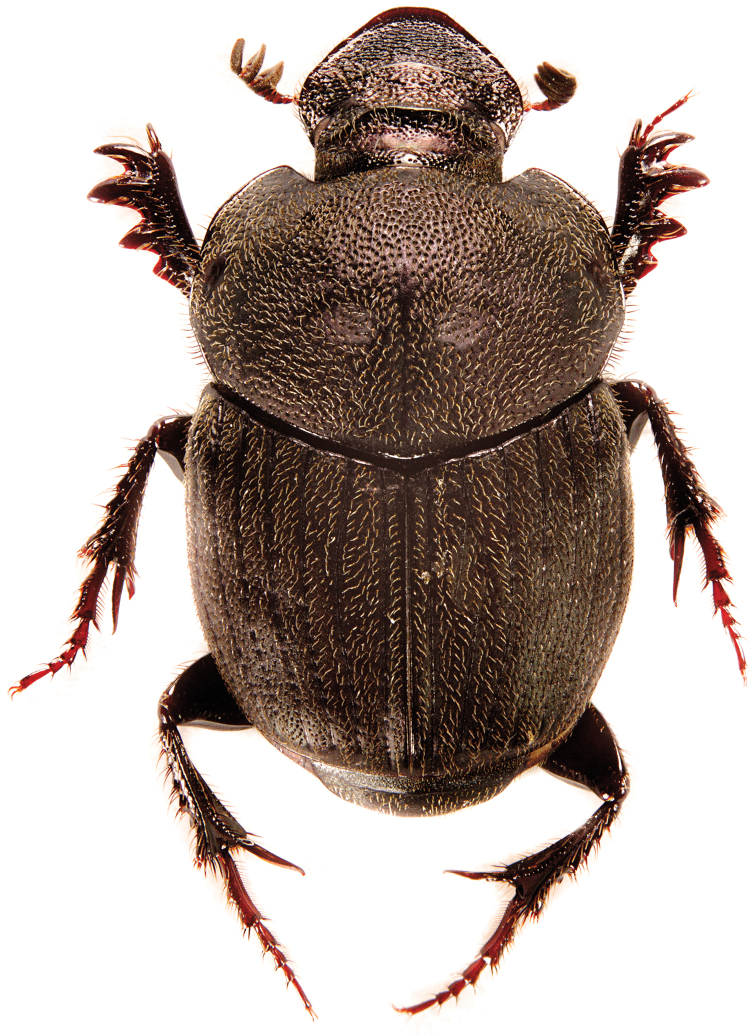
Female *Onthophagus clavijeroi* sp. n.

**Figure 4.**
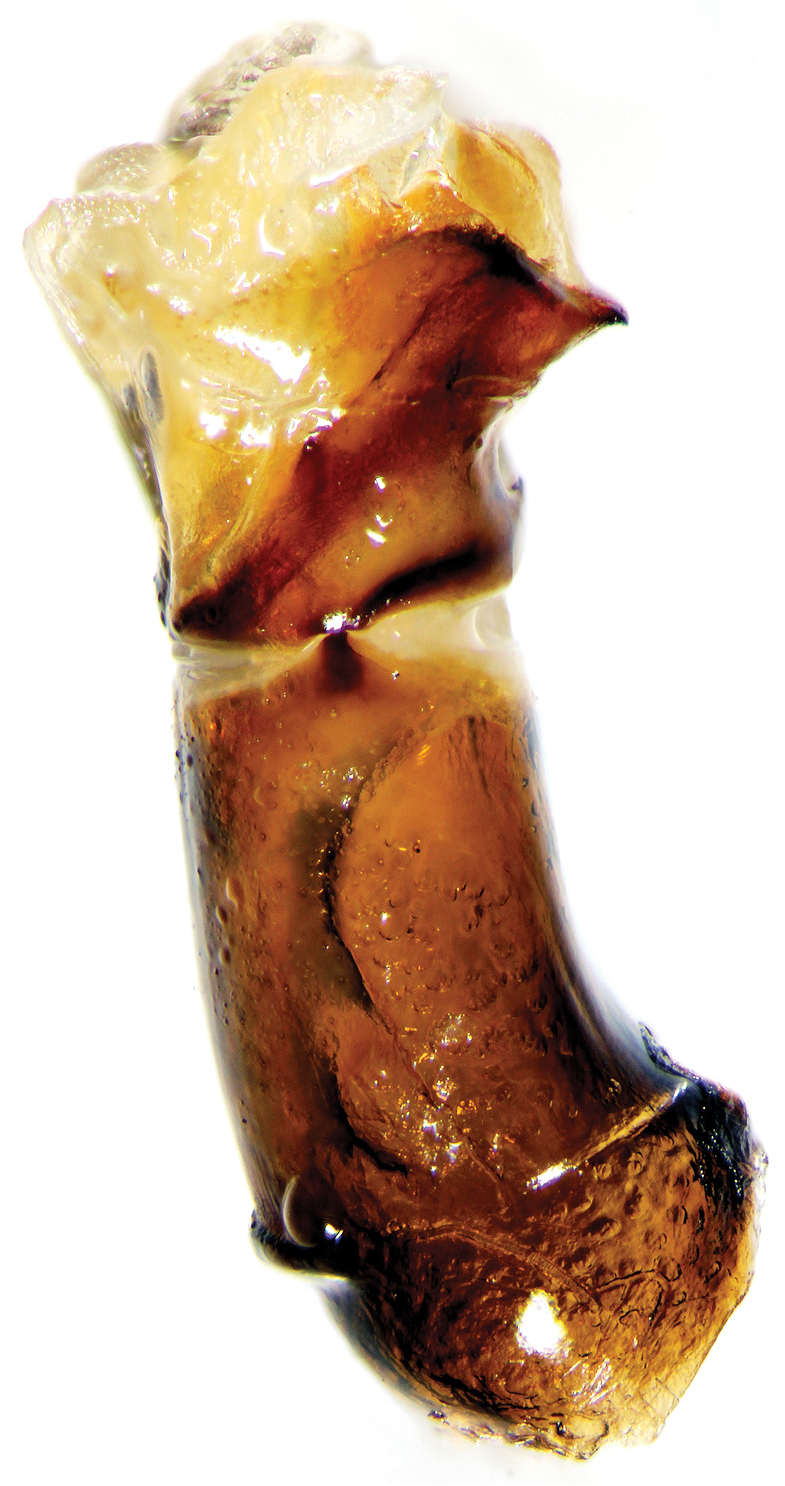
Aedeagus of *Onthophagus clavijeroi* sp. n.

**Figure 5.**
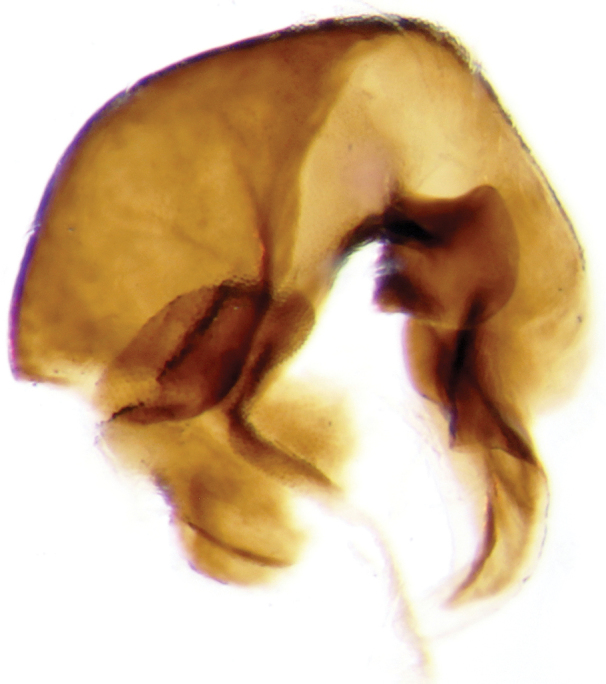
Copulatory lamella of *Onthophagus clavijeroi* sp. n.

**Figure 6.**
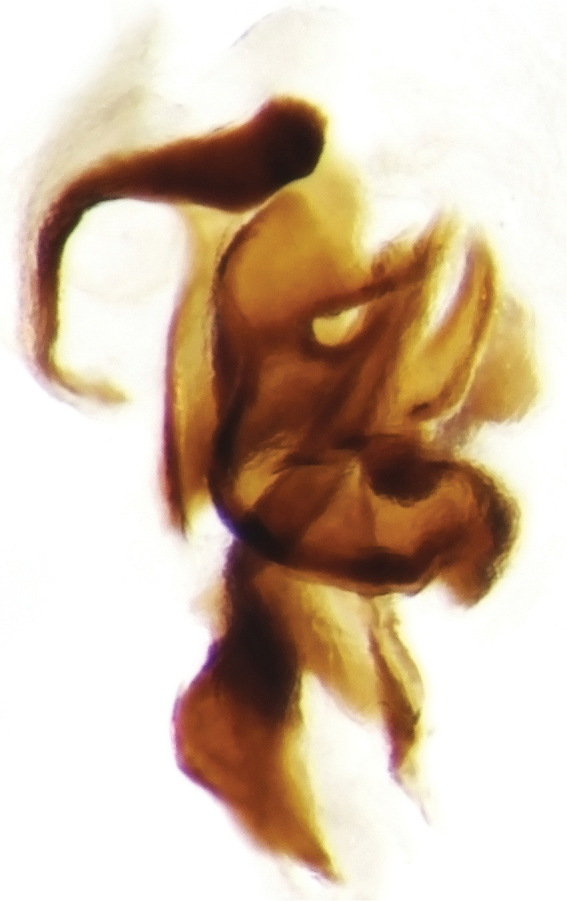
Accessory lamellae of *Onthophagus clavijeroi* sp. n.

#### Derivatio nominis.

We dedicate this species to Francisco Xavier Clavijero (Port of Veracruz, then New Spain, 9 September 1731 – Bologna, Italy, 2 April 1787), American naturalist who, together with his Chilean contemporary J. I. Molina, contested the ideas of Buffon and Pauw in their famous argument about the New World.

#### Synthesis of localities of the type material.

MEXICO: State of Puebla, El Pinal mountain, Rincón Citlaltépetl at 0.3 km from Rincón Citlaltépetl, 2704–2710 m a.s.l.; Cerro El Pinal, Santa Isabel Tepetzala at 2.5 km from Rincón Citlaltépetl, 2543 m a.s.l.

#### Type locality.

Cerro El Pinal, Rincón Citlaltépetl, state of Puebla, Mexico.

#### Type deposit.

Holotype and one paratype in the GH Collection. Six paratypes in the VM, MR, MZ and MM collections.

#### Affinities.

*Onthophagus clavijeroi* belongs undoubtedly to the *chevrolati* group (Zunino and Halffter 1988), which is well represented in the MTZ mountains, particularly in the TMVB.

Examination of the external morphology of *Onthophagus clavijeroi*, with special emphasis on the male and female genital organs, led us to consider that this new *Onthophagus* of El Pinal is closely related to the species included in the *fuscus* complex (Zunino and Halffter 1988), such as *Onthophagus fuscus* Boucomont and its subspecies (the nominotypic one, *Onthophagus fuscus mycetorum* Zunino & Halffter, *Onthophagus fuscus parafuscus* Zunino & Halffter and *Onthophagus fuscus canescens* Zunino & Halffter), *Onthophagus pseudofuscus* Zunino & Halffter and *Onthophagus semiopacus* Harold. The morphological pattern shared among these species appears to support their monophyletic origin, but a profound revision of the *fuscus* complex is required in order to properly address the identity of the polytypic *Onthophagus fuscus* and the internal systematics of the complex.

The shape and orientation of the apices of the parameres of *Onthophagus clavijeroi* and *Onthophagus fuscus fuscus* are very similar (Fig. 4), but the general shape of the lamella copulatrix (Fig. 6), especially the shape and development of both the internal carina and right branch of the copulatory lamella, allow us to easily distinguish between the two species.

**Figure 7.**
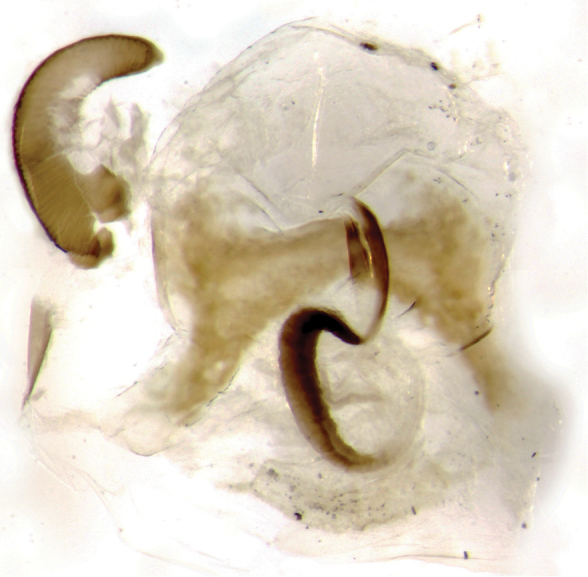
Genital apparatus of female *Onthophagus clavijeroi* sp. n.

In the female, the ventral sclerotization of the vagina corresponds to the morphological pattern already found within the *fuscus* complex, although the cephalic branches appear noticeably less developed in *Onthophagus clavijeroi* (Fig. 7).

#### Distribution and ecology

**Map 2.**
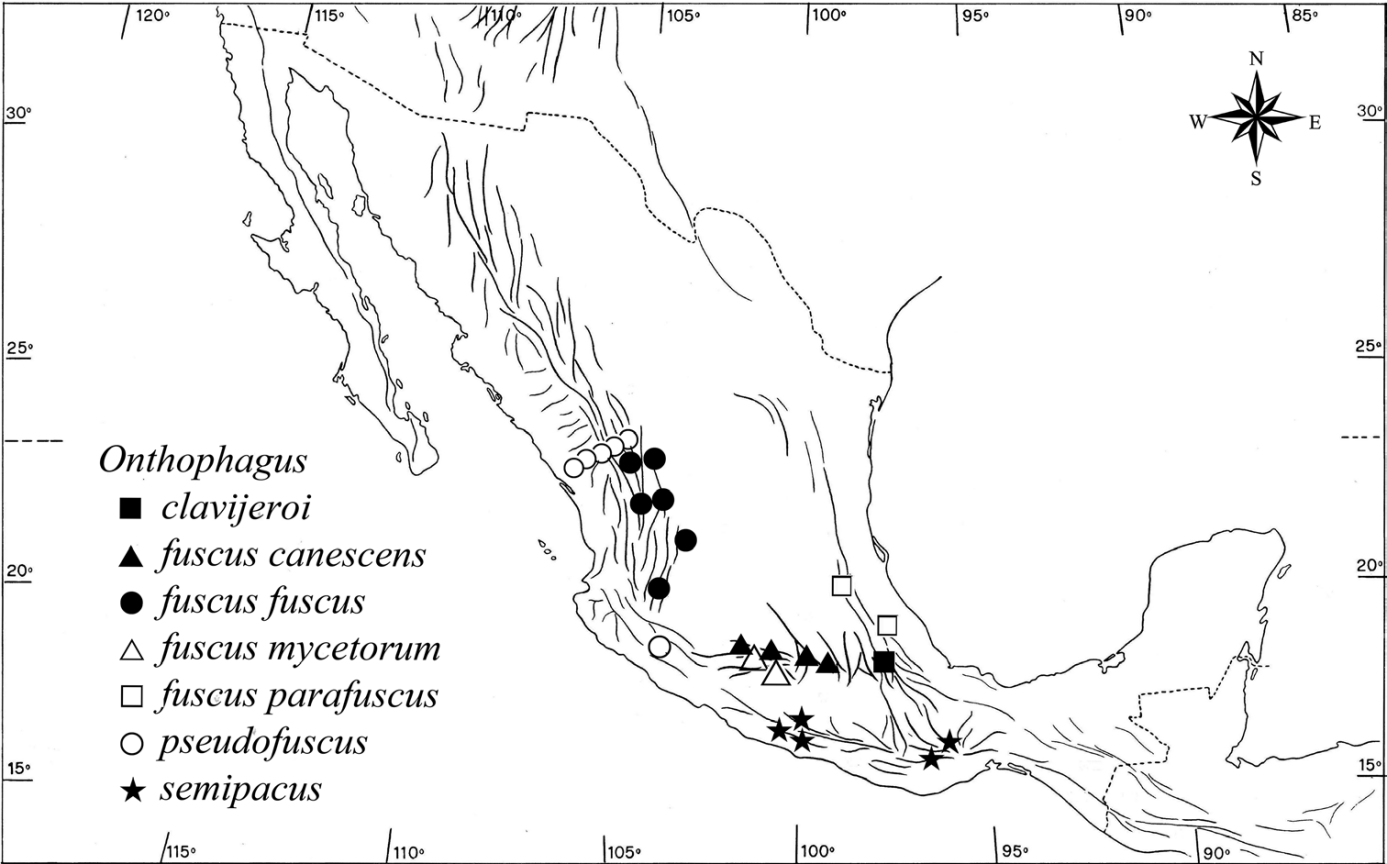
Distribution of *Onthophagus clavijeroi* and related species. Expanded from Zunino & Halffter 1988, including data from this study.

**Map 3.**
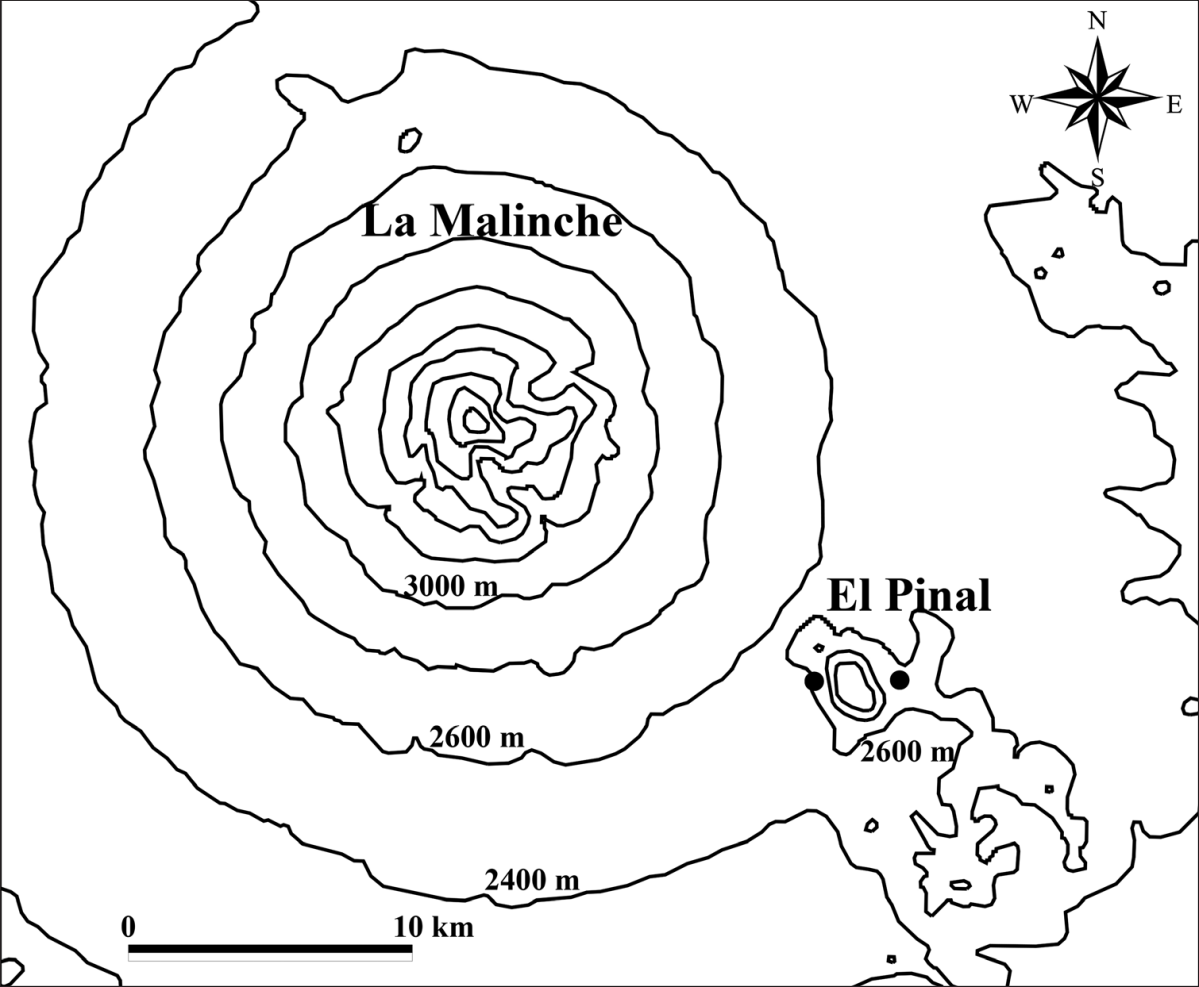
Collection sites for *Onthophagus clavijeroi* sp. n. (●). The two mountains mentioned in the text can be seen: La Malinche (4420 m a.s.l.) and El Pinal (3280 m a.s.l.).

**(Maps 2–3).** The species and subspecies of the *fuscus* complex are distributed across the mountain ranges that delimit the Mexican High Plateau, including the Sierra Madre Occidental, the TMVB and the Sierra Madre Oriental, with one species in the Puebla-Oaxaca Mountain System (Map 2). According to Zunino and Halffter (1988), the *fuscus* complex occurs to the north of the Tropic of Cancer, on the continental side of the Sierra Madre Occidental at between 2400 and 3000 m a.s.l. and following the distribution pattern of *Onthophagus fuscus fuscus*. At the same latitude, but on the Pacific side of the same mountain range, *Onthophagus pseudofuscus* is found at 2000 m a.s.l. or higher.

As noted in other groups of organisms distributed across the Sierra Madre Occidental and westernmost areas of the TMVB, the distribution of the *fuscus* complex becomes discontinuous in the proximity of the southern part of the Durango State. This interruption may be due to several factors that are not exclusive of this region, such as an interruption of geographic continuity caused by recent orographic movements, and probably the lack of beetle collection in one of the least studied mountainous areas of Mexico (to date, only one single and isolated capture of *Onthophagus pseudofuscus* has been reported for that area; see Map 2).

In the TMVB, the *fuscus* complex is represented by several subspecies of *Onthophagus fuscus*, such as *Onthophagus fuscus canescens* and *Onthophagus fuscus mycetorum* and *Onthophagus clavijeroi* (easternmost side of the TMVB). *Onthophagus fuscus parafuscus* is found in the southern part of the Sierra Madre Oriental. In addition to this U-shaped distribution across the mountain ranges that flank the Mexican High Plateau, *Onthophagus semiopacus* is found southward, in the south-eastern part of the Puebla-Oaxaca Mountain System (Map 2).

It should be noted that recent cladistic analyses conducted by Gutiérrez-Velázquez (2013, unpublished data) support the monophyly of the *fuscus* complex.

The distribution of this complex, as well as its possible monophyletic origin, the taxonomic relationships among taxa and the geomorphological history of the mountain ranges may support the hypothesis of a modern (Pleistocene to the present) distribution. To our knowledge, *Onthophagus clavijeroi* would represent a recent element within the *fuscus* complex, even considering that so far it is only known on mountains of relatively recent geological configuration.

El Pinal (3280 m a.s.l.) is associated with the mountain La Malinche (4461 m a.s.l., see Map 3) and its geological origin corresponds to the First Period of volcanic activity of La Malinche, which is estimated to have formed approximately 34 000 years ago, during the Late Pleistocene (Castro-Goeva and Siebe 2007). Further information on the Scarabaeinae of El Pinal is provided below in the Discussion.

*Onthophagus clavijeroi* was collected between 2543 and 2710 m a.s.l. in areas with primary and secondary oak forest and open habitats with shrubby vegetation and pastures. It was collected exclusively in traps baited with decaying squid. No specimens were collected in traps baited with human excrement or horse dung, nor by direct capture in other different excrement types. This implies that *Onthophagus clavijeroi* is likely to be necrophagous, a common feeding habit reported in various genera of Neotropical Scarabaeinae beetles, but one that is quite rare in American *Onthophagus*. It is possible that *Onthophagus clavijeroi* is also found in the nearby mountain of La Malinche, but systematic sampling using necrotraps remains to be carried out in that area.

### Onthophagus martinpierai

Taxon classificationAnimaliaColeopteraScarabaeidae

Moctezuma, Rossini & Zunino sp. n.

http://zoobank.org/3E8D6AE4-9280-4266-826D-72CC4C8481AB

#### Material examined.

Holotypus ♂ labeled (In Spanish) “Mexico, El Pinal, Pue., at 2.5 km from Rincón, 23/VI/13, coprotrap 5h5, x- 97°55'7.1" W, y- 19°7'48.4" N, oak-pine forest, 2530 m a.s.l., Moctezuma J.V.P. Col.”. Paratypi: 4 ♀♀, with the same label as the Holotypus; 3 ♂♂, 3 ♀♀, labeled “México, El Pinal, Pue., a 2.5 km from Rincón, 23/VI/13, coprotrap 5c5, x- 97°55'3.5" W, y- 19°7'51.8" N, oak-pine forest, 2550 m a.s.l., Moctezuma J.V.P. Col.”; 4 ♂♂, 7 ♀♀, labeled “México, El Pinal, Pue., at 2.5 km from Rincón, 23/VI/13, coprotrap 5h1, x- 97°55'13" W, y- 19°7'52.9" N, oak-pine forest, 2530 m a.s.l., Moctezuma J.V.P. Col.; 1 ♂, 1 ♀, labeled “México, El Pinal, Pue., at 2.5 km from Rincón, 23/VI/13, coprotrap 5h2, x- 97°55'11.4" W, y- 19°7'52" N, oak-pine forest, 2527 m a.s.l., Moctezuma J.V.P. Col.”; 1 ♀, labeled “México, El Pinal, Pue., at 0.3 km from Rincón, 24/VI/13, coprotrap 7c3, x- 97°54'5.8" W, y- 19°8'58.3" N, oak-pine forest, 2742 m a.s.l., Moctezuma J.V.P. Col.”; 3 ♀ ♀, labeled “México, El Pinal, Pue., at 0.3 km from Rincón, 24/VI/13, coprotrap 7h1, x- 97°53'53.9" W, y- 19°8'59" N, oak-pine forest, 2681 m a.s.l., Moctezuma J.V.P. Col.”; 2 ♀♀, labeled “México, El Pinal, Pue., at 0.3 km de Rincón, 5/VII/13, coprotrap 7h1, x- 97°53'53.9" W, y- 19°8'59" N, oak-pine forest, 2681 m a.s.l., Moctezuma J.V.P. Col.”; 2 ♀♀, labeled “México, El Pinal, Pue., at 0.3 km from Rincón, 24/VI/13, coprotrap 7h2, x- 97°53'55.8" W, y- 19°8'59.3" N, oak-pine forest, 2678 m a.s.l., Moctezuma J.V.P. Col.”; 1 ♂, 2 ♀♀, labeled “México, El Pinal, Pue., at 0.3 km from Rincón, 5/VII/13, coprotrap 7h2, x- 97°53'55.8" W, y- 19°8'59.3" N, oak-pine forest, 2678 m a.s.l., Moctezuma J.V.P. Col.”; 1 ♀, labeled “México, El Pinal, Pue., at 0.3 km from Rincón, 24/VI/13, coprotrap 7h3, x- 97°53'56.5" W, y- 19°9'1" N, oak-pine forest, 2674 m a.s.l., Moctezuma J.V.P. Col.”; 3 ♂♂, 2 ♀♀, labeled “México, El Pinal, Pue., at 0.3 km from Rincón, 5/VII/13, coprotrap 7h3, x- 97°53'56.5" W, y- 19°9'1" N, oak-pine forest, 2674 m a.s.l., Moctezuma J.V.P. Col.”; 2 ♂♂, 5 ♀♀, labeled “México, El Pinal, Pue., at 0.3 km from Rincón, 24/VI/13, coprotrap 7h4, x- 97°53'57.7" W, y- 19°9'2.4" N, oak-pine forest, 2673 m a.s.l., Moctezuma J.V.P. Col.”; 2 ♀♀, labeled “México, El Pinal, Pue., at 0.3 km from Rincón, 24/VI/13, coprotrap 7h7, x- 97°54'1.8" W, y- 19°9'6.6" N, oak-pine forest, 2684 m a.s.l., Moctezuma J.V.P. Col.”; 3 ♀♀, labeled “México, El Pinal, Pue., at 0.3 km from Rincón, 5/VII/13, coprotrap 7h7, x- 97°54'1.8" W, y- 19°9'6.6" N, oak-pine forest, 2684 m a.s.l., Moctezuma J.V.P. Col.”; 2 ♂♂, labeled “México, El Pinal, Pue., at 0.3 km from Rincón, 24/VI/13, C.D. human excrement, x- 97°53'59.3" W, y- 19°9'2.1" N, oak-pine forest, 2680 m a.s.l., Moctezuma J.V.P. Col.”.

#### Other material examined.

Mexico: Hidalgo, Metztitlán, 15 June 2005, 1♂, 1 ♀; México: Hidalgo, Metztitlán, MSN, 22 June 2005, Verdú et al. col., 3♀♀; México: Hidalgo, Metztitlán, MSN, 27 June 2005, 7♀♀.

#### Description.

**Figure 8.**
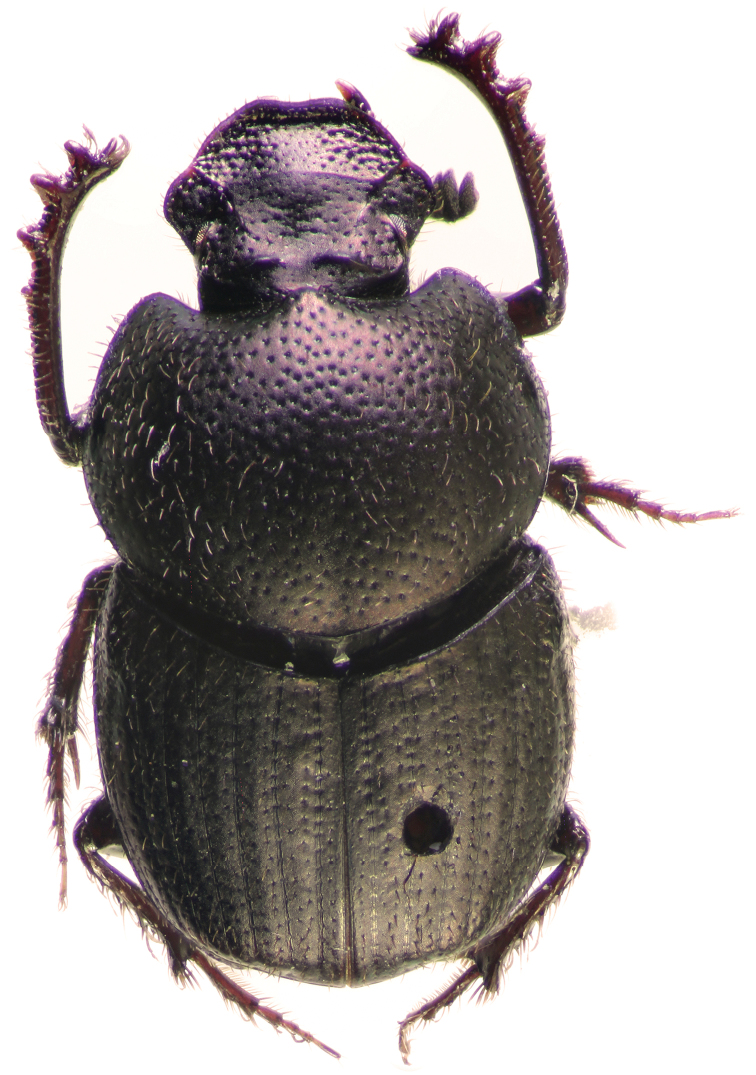
*Onthophagus martinpierai* male sp. n.

Holotype – Major male (Fig. 8). Length 5 mm, maximum width of pronotum 2.3 mm. Body black, dull and silky with cupreous-bronze casts, antennal club black. Clypeal margin distinctly sinuate and slightly reflexed at the apex, head with lateral margins clearly sinuate in proximity of clypeo-genal suture, genal margin strongly widened and rounded, genal sutures evident. Clypeal carina feeble and evenly curved, frons with two aligned and transverse carinae clearly elevated and medially separated. Clypeal surface mostly flattened, slightly depressed near anterior margin, punctures fine and shallow on clypeal disc, strong and deeper near anterior margin, fronto-clypeal surface with scattered and irregular punctures, genae with few and stronger punctures, head surface with reticular microsculpture, some punctures are associated with short and yellowish setae.

Pronotum completely bordered, lateral and posterior margins evenly curved, anterior and superior side with a conical, tubercle-like protuberance. Pronotum with deep and setigerous punctures, some of them associated with a small and rounded granule on anterior margin, especially in proximity of anterior angles of pronotum; punctures less impressed near the posterior margin, reticular microsculpture evident. Elytral striae impressed, with simple to medium-sized punctures quite separated, interstriae almost flat, with small punctures associated with very tiny granules, less evident in females; same reticular microsculpture of pronotum. Pygidium with strong and dense punctures, punctuation smaller, shallower and less dense near base, pygidial surface with fine microsculpture, which is less evident at apex where pygidium appears shinier. Protibiae slender, elongated and apically strongly curved inward, inner margin wider at the apex, inner and apical angle with tuft of long, robust and yellowish setae; protibia with four external teeth well separated and distributed along apical half, external margin and intervals between external teeth serrated. Parameres and endophallic sclerites: Figs 10–12.

Variation. Minor male: Smaller than major males, clypeus more clearly trapezoidal, clypeal carina more evident, pronotal protuberance either absent or feebly indicated, foretibiae less elongated and curved at the apex, similar to those of the female.

**Figure 9.**
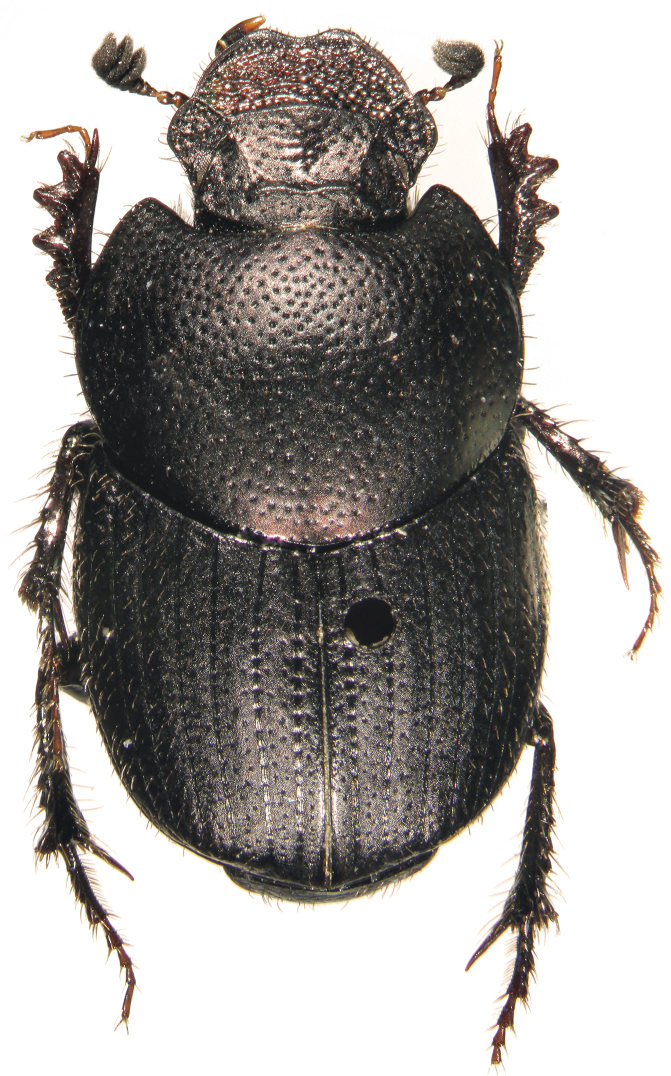
Female *Onthophagus martinpierai* sp. n.

Female (Fig. 9): Clypeus distinctly trapezoidal shaped and wider, clypeo-genal suture indicated by feeble depression on lateral margin of head, genae narrower, clypeal carina distinct and stronger than in male, frons with transversal carina slightly but clearly depressed at middle, clypeal punctuation stronger and evenly distributed. Pronotal protuberance either absent or very feebly indicated, protibiae shorter than in male and wider at apex, inner angle of the protibiae without tuft of setae, with few erected setae at most. Female genitalia: Fig. 13.

**Figure 10.**
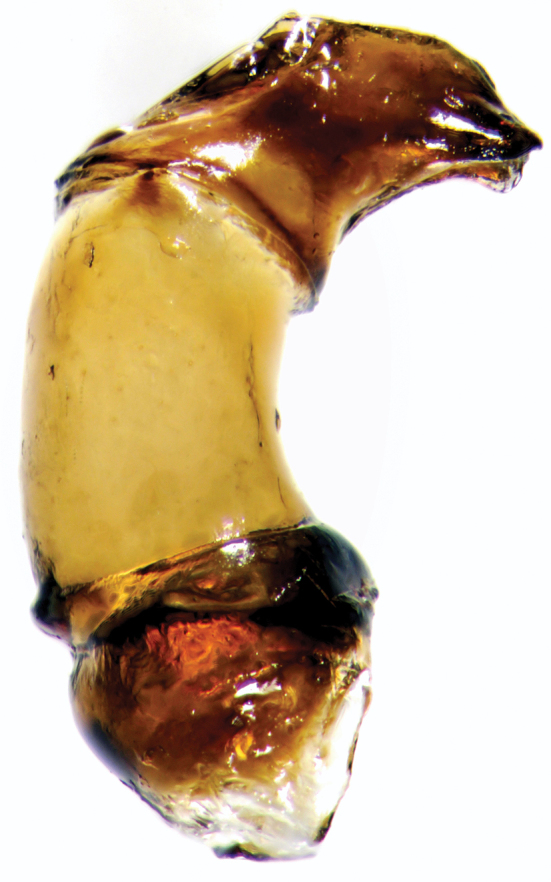
Aedeagus of *Onthophagus martinpierai* sp. n.

**Figure 11.**
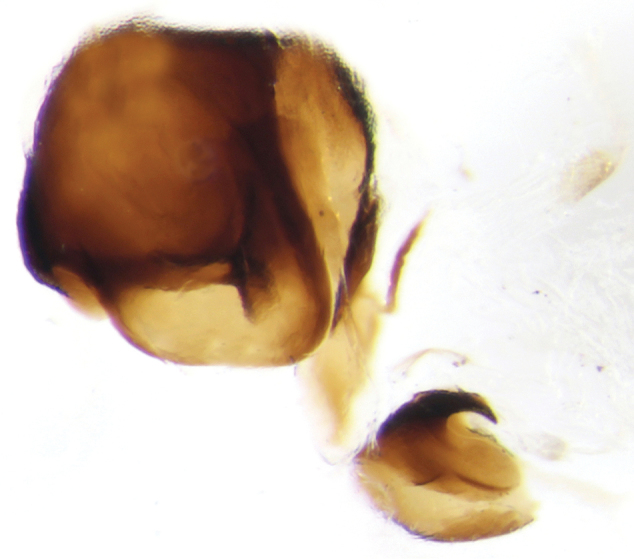
Copulatory lamella of *Onthophagus martinpierai* sp. n.

**Figure 12.**
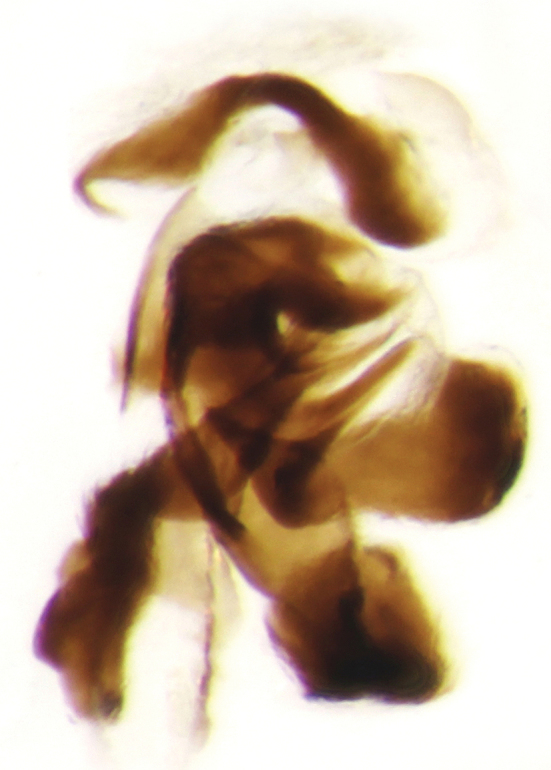
Accessory lamellae of *Onthophagus martinpierai* sp. n.

**Figure 13.**
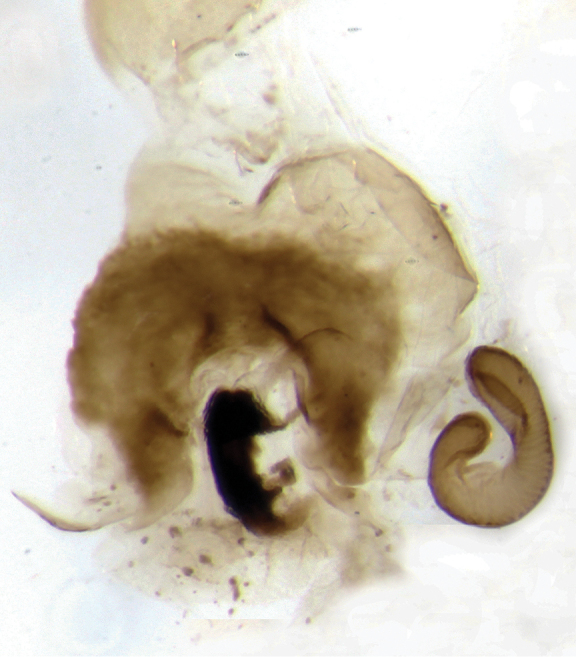
Genital apparatus of female *Onthophagus martinpierai* sp. n.

#### Derivatio nominis.

We dedicate this new species to Fermín Martín Piera (Madrid, Spain, 7 July 1954–ibid., 19 July 2001), entomologist and ecologist, who rescued modern Scarabaeoidology in Spain in the 1980s.

#### Synthesis of the localities of the type material.

MEXICO: State of Puebla, El Pinal mountain (Rincón Citlaltépetl) at 0.3 km from Rincón Citlaltépetl, 2673–2742 m a.s.l.; El Pinal mountain (Santa Isabel Tepetzala) at 2.5 km from Rincón Citlaltépetl, 2530–2550 m a.s.l.

#### Type locality.

El Pinal mountain, Santa Isabel Tepetzala, state of Puebla, Mexico.

Type repository: Holotype and five paratypes in the GH Collection. Paratypes in the collections of VM, MR, MZ, MM, CNC and LD collections.

#### Affinities.

The morphology of *Onthophagus martinpierai* led us to include this new species of El Pinal in the *landolti* group (Zunino and Halffter 1997). This group is widely distributed from the southern border of Ontario (Canada) to the central regions of Amazonia (MR, unpublished data), with the highest diversity found in North America. To our knowledge, the *landolti* group may include several complexes of species (e.g. *lecontei-subopacus* complex, Howden and Génier 2004), the taxonomy and systematics of which require a profound revision.

This new species shares significant diagnostic characters, possible synapomorphic traits, with *Onthophagus dubitabilis* Howden & Génier and *Onthophagus mariozuninoi* Delgado, Navarrete & Blackaller. These include the shape of the frontal carina in the male, shape of the parameres (Fig. 10) and the shape of the copulatory lamella (the latter has not been published for *Onthophagus mariozuninoi*) (Fig. 11).

#### Distribution and ecology

**Map 4.**
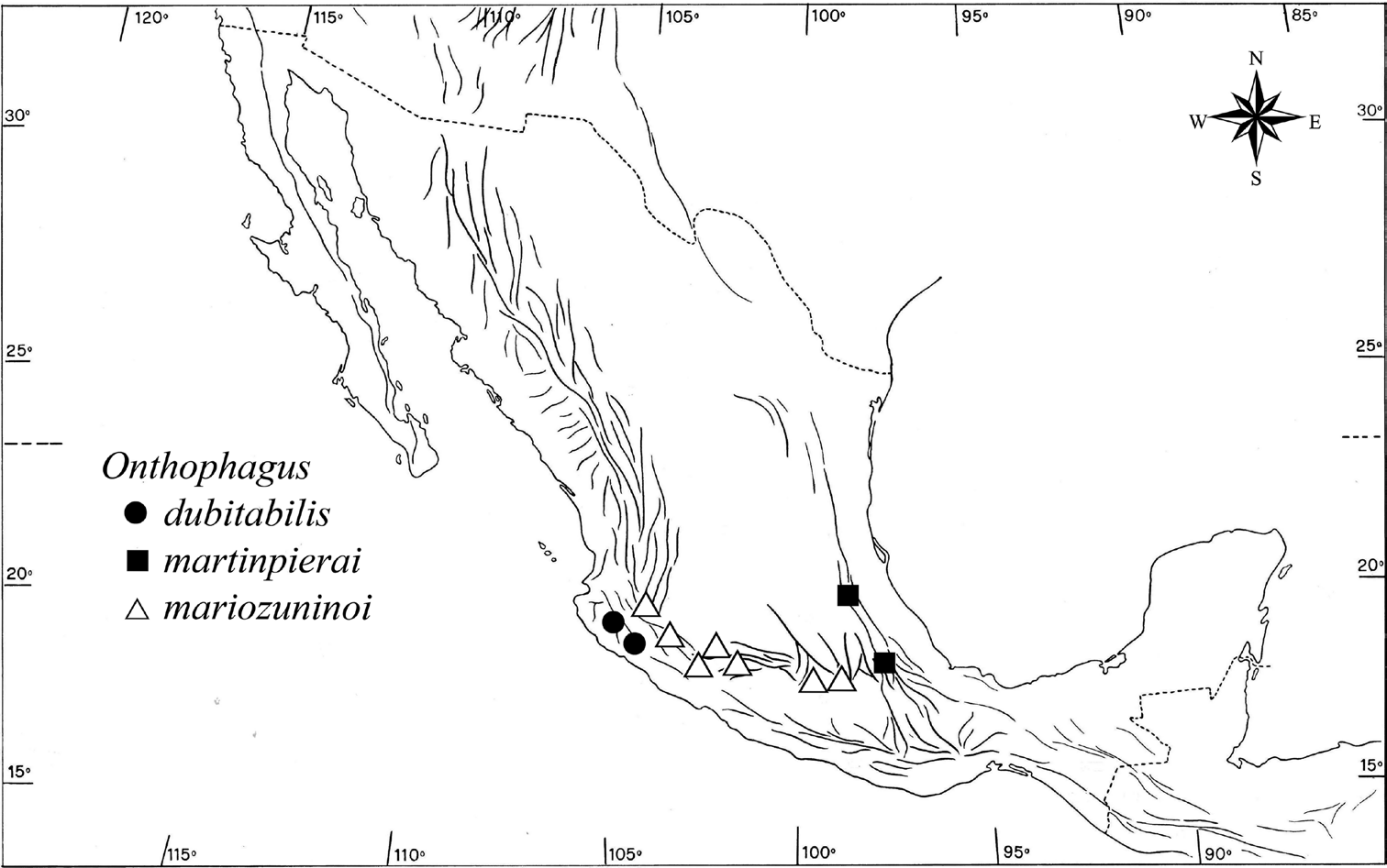
Distribution of *Onthophagus martinpierai* and related species.

**Map 5.**
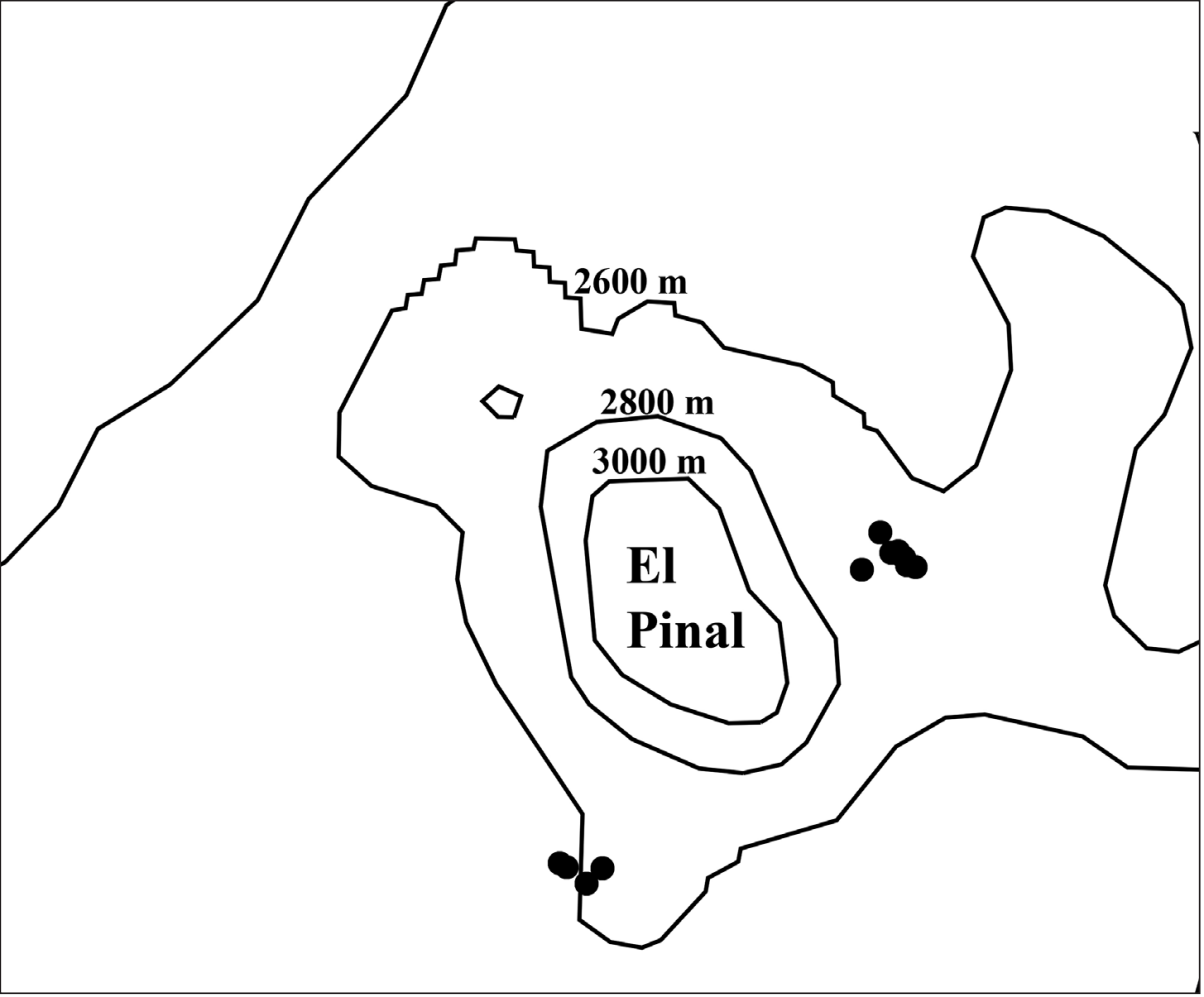
Collection sites for *Onthophagus martinpierai* (●) on the mountain El Pinal.

**(Maps 4–5).** *Onthophagus martinpierai*, *Onthophagus dubitabilis* and *Onthophagus mariozuninoi* seem to represent a new complex of species within the *landolti* group, occurring from the southern mountain range of the Sierra Madre Occidental and the nearby Sierra Madre del Sur, across the entire TMVB. As has been observed and proposed for the *fuscus* complex of the *chevrolati* group, these three species appear to show a modern distribution centred in the TMVB, with the occurrence in the southern Sierra Madre Occidental and eastern TMVB representing a secondary and more recent expansion of these species.

*Onthophagus mariozuninoi* is distributed from the south-eastern Sierra Madre Occidental (Tequila, Jalisco) and south-western TMVB (Atenquique, Jalisco) to the central-eastern part of the TMVB (Tlayacapan, Morelos) (Map 4). We provide Patzcuaro and Morelia, Michoacan, as geographic data additional to the known distribution of *Onthophagus mariozuninoi* (Delgado et al. 1993, Delgado 1997, Howden and Génier 2004).

*Onthophagus dubitabilis* has been described by Howden and Génier (2004) for Autlán, Jalisco. We add two new records for this species: El Tapeiztle, Minatitlán Municipality, Colima, 2300 m a.s.l., pasture, 14-VIII-1989. L.E. Rivera col., 1 male; Laboratorio Natural Las Joyas de Manantlán, Sierra de Manantlán mountains, Jalisco, 8-IX-1987, L.E. Rivera and V. Bedoy col., 1 female. These new localities, along with that provided by Howden and Génier (2004), are located in the mountainous region of the Sierra de Manantlán, which is located in the south-western part of Jalisco and northern region of Colima. The Sierra de Manantlán forms part of the Sierra Madre del Sur.

*Onthophagus martinpierai* has been found in El Pinal, in the easternmost part of the TMVB, the same locality as *Onthophagus clavijeroi*. However, it is very likely this species also occurs in other localities across the central-eastern part of the TMVB (eastern limit of *Onthophagus mariozuninoi*’s distribution).

*Onthophagus martinpierai* has been collected both directly and using pitfall traps baited with human excrement and horse dung, The large number of specimens collected mainly with excrement suggests the coprophagous habit of this species. It was found in areas with secondary pine-oak forests with pastures between 2530–2742 m a.s.l.

In contrast, *Onthophagus mariozuninoi* appears to be generalist since it has been collected in dog scats and pitfall traps baited with squid, even though the largest number of individuals has been captured on fungi. This species seems to occur between 1650–2370 m a.s.l. In Pátzcuaro, Michoacán, it was collected at 2150 m a.s.l. According to Delgado et al. (1993), this species is eurytopic and is found in mountain mesophilous and pine-oak forests. In Pátzcuaro, it was also collected in pine-oak forest.

The Sierra de Manantlán mountain range was originally a forested area, presenting distinct vegetation at different altitudes. *Onthophagus dubitabilis* has been collected in both mountain mesophilous forests and open habitats at between 1900–2300 m a.s.l.

## Results and discussion

The Scarabaeinae beetles collected during our fieldwork in El Pinal belong to the genus *Onthophagus*. In addition to *Onthophagus clavijeroi* and *Onthophagus martinpierai*, five more species have been found in the same localities: *Onthophagus chevrolati retusus* Harold, *Onthophagus incensus* Say, *Onthophagus lecontei* Harold, *Onthophagus mexicanus* Bates, and *Onthophagus bolivari* Moctezuma, Rossini & Zunino (a new species recently described in Arriaga et al. 2016).

As proposed at the beginning of this paper (prediction 1), species that occur above 2500 m belong to the Paleo-American Distribution Pattern. In the MTZ, the genus *Onthophagus* is a lineage of ancient penetration, showing high diversity in terms of both number of species and eco-geographic distribution. These characteristics can explain the ability of the genus to fit the Palaeo-American pattern in different ways. Only one specimen of *Onthophagus incensus* (a species very abundant at lower altitudes) has been collected at 2639 m. This species is distributed from Mexico to the northern regions of South America (Colombia and Venezuela) and associated with tropical conditions, although it also occurs in mountainous areas up to 2000 m a.s.l. This specimen was collected on the lowest part of El Pinal, in the same sampled area in which a large number of *Onthophagus mexicanus* was found, a species widely distributed on the Mexican High Plateau.

Among the lineages of *Onthophagus* (see Zunino and Halffter 1997) distributed across the southern High Plateau and foothills of the surrounding mountain ranges (Paleo-American Distribution on the High Plateau), two species have been found: *Onthophagus lecontei* (*landolti* group) and *Onthophagus mexicanus* (*mexicanus* group) (Zunino and Halffter 1997).

All of the remaining species, which constitute common elements of the scarabaeinae fauna of the TMVB, belong to the Mountain Paleo-American Distribution Pattern. *Onthophagus chevrolati retusus* is a very characteristic subspecies of the TMVB, reaching the highest elevation to date reported for Scarabaeinae in the MTZ (see Zunino and Halffter 1988). Indeed, this subspecies was found in the highest sampled locality of El Pinal (3017 m). *Onthophagus chevrolati retusus* occurs in the eastern part of the TMVB, both in the Sierra Madre del Sur and in the Puebla-Oaxaca Mountain System.

*Onthophagus clavijeroi* and *Onthophagus martinpierai*, along with closely related species, occur in the central and western parts of the TMVB, a distribution pattern shared with *Onthophagus bolivari* (Arriaga et al. *in press*), the most abundant species of El Pinal, which was also collected on the mountain La Malinche.

El Pinal, a relatively recent and not particularly large mountainous formation, thus hosts representatives of the three main biogeographic patterns to which the genus *Onthophagus* corresponds across the MTZ. However, on older and higher mountains on the eastern side of the TMVB, the Mountain Palaeo-American distribution pattern will dominate above 2500 m.

Concerning prediction 2, it is important to emphasize that the species collected on El Pinal, as well as their close relatives, can be also found in western and eastern localities of the TMVB, including the Sierra Madre Oriental. As far as we could ascertain in this study, El Pinal appears to have been colonized from both the western and eastern sides, apart from with *Onthophagus lecontei* and *Onthophagus mexicanus*, which possibly arrived via vertical dispersion from the contiguous Mexican High Plateau. Instead, the remaining species (including *Onthophagus incensus*) appears to have adopted a horizontal distribution from the lower altitudes of the eastern TMVB.

The high level of endemism observed on El Pinal, quite unexpected on such a relatively small and recent mountain, may be the result of a recent vicariant event or due to the lack of intensive sampling in the central mountains of the TMVB and the nearby Puebla-Oaxaca Mountain System.

As indicated above, prediction 3 has been fully met, as has prediction 5. We could not test the fourth prediction because of the isolation of El Pinal on the northern side of the TMVB, as well as its lack of contact with the southern tropical depressions.

In accordance with the sixth preliminary hypothesis, the highest value of abundance and number of species were found at 2639 m. In contrast, sampling carried out at higher altitudes (2993 m) yielded a distinctly lower number of species and individuals: *Onthophagus chevrolati retusus* and *Onthophagus bolivari* were the only species of the *chevrolati* group to be collected, with a few specimens (e.g., only one individual for *Onthophagus bolivari*). This altitudinal gradient firstly led us to suppose that some species and subspecies of the *chevrolati* group can reach the highest altitudes in the Mexican mountains. In other mountains of the TMVB, even *Onthophagus hippopotamus* Harold, a species of the *chevrolati* group associated with gopher nests, occurs at high elevations. Secondly, it is important to note that the altitudinal range between 3000–3100 m a.s.l. represents, with extreme rarefaction, the upper distributional limit of the mountain Scarabaeinae in the MTZ.

Regarding the seventh preliminary hypothesis, the *Onthophagus* of El Pinal are found in different types of mountain vegetation (e.g., opened and secondary forests, clearings etc.), with greatest abundance in areas dominated by plants that require high solar radiation. Furthermore, the presence and density of trees do not appear to affect the distribution of *Onthophagus* of El Pinal, in the way that they have been proved to be determinant in the tropical forest of the ZTM.

## Supplementary Material

XML Treatment for Onthophagus clavijeroi

XML Treatment for Onthophagus martinpierai
